# Alteration of lipopolysaccharide O antigen leads to avirulence of gut-colonizing *Serratia marcescens*

**DOI:** 10.3389/fmicb.2023.1278917

**Published:** 2023-11-09

**Authors:** Junbeom Lee, Jong Uk Kim, Bok Luel Lee, Jiyeun Kate Kim

**Affiliations:** ^1^Metabolomics Research Center for Functional Materials, Kyungsung University, Busan, Republic of Korea; ^2^Host Defense Protein Laboratory, College of Pharmacy, Pusan National University, Busan, Republic of Korea; ^3^Department of Microbiology, Kosin University College of Medicine, Busan, Republic of Korea

**Keywords:** *Riptortus pedestris* (=R. Clavatus), *Serratia marcescens* Db11, entomopathogen bacteria, O antigen, insect-pathogen interaction

## Abstract

The reason why the potent entomopathogen *Serratia marcescens* fails to kill insects through oral infection is unknown. To compare effects of septic injection and oral administration of *S. marcescens*, we used a model bean bug, *Riptortus pedestris*. Most *R. pedestris* insects survived oral infections, but not septic infections. Although the number of *S. marcescens* cells in hemolymph after oral infection, which were originated from gut-colonizing *S. marcescens*, was higher than the fatal number of cells used in septic injection, they did not kill host insects, suggesting a loss of virulence in gut-colonizing *S. marcescens* cells. When gut-colonizing *S. marcescens* cells were septically injected into insects, they failed to kill *R. pedestris* and survive in hemolymph. To understand the avirulence mechanisms in gut-colonizing bacteria, lipopolysaccharides of *S. marcescens* were analyzed and revealed that the O antigen was lost during gut colonization. Gut-colonizing *S. marcescens* cells were resistant to humoral immune responses but susceptible to cellular immune responses, easily succumbing to phagocytosis of hemocytes. When cellular immunity was suppressed, the gut-colonizing *S. marcescens* cells recovered their virulence and killed insects through septic injection. These results suggest that a key mechanism of avirulence in orally infected *S. marcescens* is the loss of the O antigen, resulting in susceptibility to host’s cellular immune responses.

## Introduction

*Serratia marcescens* is a gram-negative bacterium belonging to the family Yersiniaceae that is present in various environments. It infects a wide range of hosts, including mammals, invertebrates, and plants (Hejazi and Falkiner, 1997). *S. marcescens* is a potent entomopathogen and is known to produce insecticidal molecules that cause bacteremia in insects, bleeding of hemolymph, and rapid killing of insects (Chadwick et al., 1990; Ishii et al., 2012). The *S. marcescens* Db11 strain, which uses a septic infection route, is highly pathogenic to *Drosophila melanogaster*, *Apis mellifera* insect and *Caenorhabditis elegans* nematode (Flyg et al., 1980; Kurz et al., 2003; Raymann et al., 2018). In fruit flies, host survival is dramatically reduced by septically injecting 50–100 *S. marcescens* cells (Nehme et al., 2007). *S. marcescens* Db11 cells can colonize the midguts of the fruit fly *D. melanogaster* and bean bug *Riptortus pedestris* (Hemiptera: Alydidae) through an oral infection route without inducing toxicity (Nehme et al., 2007; Lee and Lee, 2022). These reports suggest that the pathogenicity of *S. marcescens* varies by the route of infection (orally or directly). This study was conducted to reveal the molecular mechanisms responsible for the avirulence of *S. marcescens* cells that infect a host via the oral route.

Recent studies on the interactions between *S. marcescens* and bean bug *R. pedestris* show that *S. marcescens* cells are resistant to isoforms of the antimicrobial peptide thanatin in the hemolymph and to trialysin in the salivary fluid of *R. pedestris* (Lee D.J. et al., 2017; Lee et al., 2022). In addition, *S. marcescens* suppresses the expression of serralysin toxins during colonization of the M3 midgut of *R. pedestris* (Lee and Lee, 2022). Although these results partly explain how orally infected *S. marcescens* survives and is less toxic in *R. pedestris*, we speculated that there is a more critical reason why *S. marcescens* cells lose their virulence upon oral infection of the host. To investigate the molecular mechanisms of avirulence in orally infected *S. marcescens*, gut-colonizing *S. marcescens* cells were collected from the M3 midgut and examined for their virulence to *R. pedestris*. Lipopolysaccharide (LPS) samples taken from M3-colonzing *S. marcescens* were analyzed, and the effects of LPS alteration in *S. marcescens* cells were further examined with respect to the susceptibility to humoral and cellular immunity of *R. pedestris*.

## Materials and methods

### Bacteria and media

Non-pigmented *S. marcescens* strain Db11 is a spontaneous streptomycin-resistant mutant of Db10, which was originally isolated from a moribund fly (Flyg et al., 1980). *S. marcescens* strain 20C2 is a Db11 miniTn5Cm insertion mutant showing O antigen deficiency (Kurz et al., 2003). Green fluorescent protein (GFP)-labeled *S. marcescens* is Db11 transformed with plasmids expressing GFP (Nehme et al., 2007). These strains were cultured to mid-log phase at 37°C in Luria-Bertani medium (LB; BD Difco, United States) with the appropriate antibiotics such as 100 μg/mL streptomycin and 100 μg/mL ampicillin (Lee D.J. et al., 2017).

### Insect rearing and inoculation of *Serratia marcescens* cells

*R. pedestris* insects were maintained in insect laboratory at 28°C under a daily cycle of 16 h of light and 8 h of dark, as described previously (Kim et al., 2013, 2014, 2017; Lee et al., 2015, 2019, 2020, 2023; Lee J. et al., 2017; Jeong et al., 2023; Lee and Lee, 2023). Nymphal insects were reared in clean plastic containers with soybean seeds and distilled water containing 0.05% ascorbic acid (DWA). When newborn males molted to the adult stage, *S. marcescens* cells in an inoculum solution were provided with wet cotton balls in a small petri dish. The inoculum solution consisted of mid-log phase *S. marcescens* cells in DWA at a concentration of 10^7^–10^9^ cells/mL.

### Septic injection of bacteria in *Riptortus pedestris*

*S. marcescens* cells were washed with 10 mM phosphate buffer (PB; pH 7.0; Gibco, United States) and suspended in Grace’s insect medium (Gibco, United States) to produce different bacterial colony-forming units (CFUs) at 20–300 CFU/μL. Two microliters of the bacterial cell solution were injected into male *R. pedestris* 3 days after molting into the adult stage using a glass capillary and an air-pressure injector (Picospritzer, Parker, United States). Survival rates were monitored every 6 h after bacterial septic injection.

### CFU assays for *Serratia marcescens* titers in M3 midguts and hemolymph after oral infection

Cultured *S. marcescens* cells were washed with PB and resuspended in Grace’s insect medium at a density of 2.0 × 10^8^ cells/mL. Each male *R. pedestris* was infected orally with 10 μL of bacterial solution, and CFUs in M3 and hemolymph were measured at one-day intervals. Individual M3 midguts dissected from *R. pedestris* were collected in 100 μL of PB, homogenized using a plastic pestle, and serially diluted with PB (Lee and Lee, 2022). A decoagulation buffer (15 mM NaCl, 30 mM trisodium citrate, 26 mM citric acid, and 20 mM EDTA, containing 5% glycerol, pH 5.0) was used to collect 2 μL of hemolymph by cutting the legs. The collected solution was filtered through a mesh with a pore size of 0.2 μm (Ultrafree-MC, Millipore, United States) and plated on streptomycin-containing LB agar plates (100 μg/mL). The plate colonies were counted after 1 day of incubation at 37°C. The number of CFUs per insect was calculated by multiplying the colony count by the dilution factor.

### CFU assay for *Serratia marcescens* titers in whole bodies after septic injection

To detect *S. marcescens* cells in the entire body of the insects, male *R. pedestris* were septically injected with 50 cells of cultured *S. marcescens* or M3-colonizing *S. marcescens* (Kim et al., 2015b). Each *R. pedestris* insect was immersed in 200 μL of PB with their legs cut off. After vigorous vortexing, the solution was collected using a syringe and transferred to a new tube. The collected solution was filtered through a mesh with a pore size of 5.0 μm (Millex-SV, Millipore, United States), serially diluted, and plated on streptomycin-containing LB agar plates to count the CFUs.

### Isolation of M3-colonizing *Serratia marcescens* cells

*R. pedestris* M3 midguts colonized with orally fed *S. marcescens* cells were dissected under a microscope, placed in 50 μL of PB, and then cut into pieces with fine scissors to release bacteria from the midguts (Lee and Lee, 2022). One milliliter of PB was mixed with the M3 pieces by gentle pipetting to spill the *S. marcescens* cells to the solution. The solution was then filtered through a 5.0 μm pore to remove the gut tissues. *S. marcescens* cells were washed with PB to remove the host molecules.

### Purification of LPS

LPS was extracted from fully grown bacterial cells using a modified hot-phenol method (Westphal, 1965). Fully grown *S. marcescens* cells (10^9^) were washed with PB and resuspended in 500 μL of PB. An equal volume of hot phenol was added to the cell solution and incubated in a water bath at 65°C. The cell solution was vortexed rigorously every 5 min. After 1 h of incubation, the solutions were cooled and 200 μL of chloroform was added. After vortexing, the solution was incubated at room temperature for 5 min and then centrifuged at 15,000 × *g* for 15 min to separate the water and phenol phases. Next, 400 μL of the water-phase solution was transferred to a new tube and 800 μL of isopropanol was added to the solution. After allowing the LPS to precipitate at −20°C overnight, the precipitates were collected by centrifugation at 20,000 × *g* for 20 min, washed with 80% ethanol, and air dried. The extracted LPS precipitates were used to purify O antigens.

### Generation of polyclonal antibodies against O antigens of *Serratia marcescens*

To separate and collect O antigens from *S. marcescens* LPS, LPS samples were solubilized (5 mg/mL) in 2% acetic acid and incubated for 2 h at 100°C. The hydrolyzed LPS samples were cooled to room temperature and subjected to centrifugation for 10 min at 8,000 × *g*. The supernatants were carefully collected and lyophilized to concentrate the O antigens. To further purify the O antigens, lyophilized O antigen samples were solubilized (10 mg/mL) in pure water and subjected to centrifugation for 10 min at 8,000 × *g*, and the supernatants were filter-sterilized in 0.22 μm pore. Finally, the purified O antigens were lyophilized, and their final weights were measured.

To prepare glycoconjugates for antibody generation, the *S. marcescens* O antigens were solubilized at 2.5 mg/mL in 0.1 M sodium meta-periodate (pH 5.5) and incubated for 30 min at room temperature in the dark. To remove oxidizing agents, the O antigen samples were applied to Zeba Desalt spin columns (Pierce, United States) equilibrated with PBS, and the eluate was collected (Brett et al., 2011). To each milliliter of the O antigen solution, 125 μL of the keyhole limpet hemocyanin carrier protein (10 mg/mL) (Sigma, United States) in PBS was added. Following mixing with gentle agitation, 10 μL of 5 M sodium cyanoborohydride (Pierce, United States) was added to each milliliter of the conjugation mixture, and the reaction mixture was incubated overnight at room temperature in the dark. To remove the reducing agents, the conjugation mixture was run through a Zeba spin desalting column equilibrated with PBS, and the eluate was collected. Using the O antigen-conjugated carrier proteins, we raised rabbit anti-*Serratia* O antigen polyclonal antibodies using a standard immunization protocol.

### Western blots of LPS O antigens

For immunoblot analyses, 10^7^ cultured *S. marcescens* and M3-colonizing *S. marcescens* cells were collected in 10 mM PB and washed. Samples were subjected to tricine sodium dodecyl sulfate polyacrylamide gel electrophoresis (SDS-PAGE) with 12% gels and transferred to a polyvinylidene difluoride membrane (Lee D.J. et al., 2017; Lee and Lee, 2022). After blocking with 10% skim milk in a Tris buffered saline with Tween 20 (TBS-T) for 1 h, the membrane was incubated at room temperature for 1 h with anti-*Serratia* O antigen antibody. The membranes were washed six times with TBS-T buffer and visualized with horseradish peroxidase-conjugated goat anti-rabbit immunoglobulin G secondary antibody (dilution factor 1:10,000; Santa Cruz, United States) for 1 h.

### Bacterial susceptibility assay against host immune responses

Immune responses were induced in fifth-instar *R. pedestris* nymphs by septically injecting PB or *Escherichia coli* solution (10^8^ cells/mL). After 24 h, 100 μL of hemolymph was collected in 100 μL of decoagulation buffer or PB. The heat-treated hemolymph was used for the susceptibility assay of the humoral immune response after centrifugation at 20,000 × *g* for 10 min. The protein concentration of hemolymph was determined using a Bradford assay (Kruger, 1994). For the susceptibility assay to humoral immune response, 500 *S. marcescens* cells were treated with 100 μL of heat-treated hemolymph (final concentration = 10 μg/mL) at 37°C for 2 h and plated on streptomycin-containing LB agar plates to measure CFUs. For the cellular immune response assay, *S. marcescencs* Db11 strain expressing GFP was used to determine the phagocytosis of *R. pedestris* hemocytes. To inhibit phagocytosis of insect immune cells, a latex bead solution (FluoSpheres^®^ Fluorescent Microspheres, Invitrogen, United States) was injected prior to bacterial injection as described previously (Kim et al., 2015a). Beads were washed with PB and suspended with Grace’s insect medium to create a solution containing 0.9 × 10^13^ beads/mL. Two microliters of the bead solution were injected into male *R. pedestris* 1 day prior to the bacterial challenge. To examine hemocyte phagocytosis, hemolymph was collected 3 h after injection of the bacteria (4.0 × 10^5^ CFUs per insect). The rate of phagocytosis was determined using a fluorescence microscope (AX70, Olympus, Japan) (Metabolomics Research Center for Functional Materials, Kyungsung University, South Korea) at 400 × magnification, and insect survival was monitored every 6 h.

### Statistical analyses

The statistical significance of the survival rate was determined using Log-rank (Mantel-Cox) test, as provided in GraphPad Prism (ver. 8.0; GraphPad Software, United States). The statistical significance of the CFUs data was determined using an unpaired multiple *t-*test.

## Results

### Orally infected *Serratia marcescens* cells were recovered from both host midgut and hemolymph

**Figure 1:**
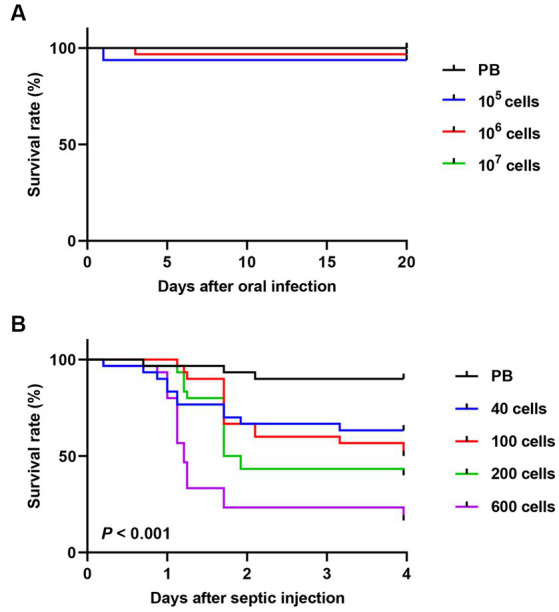
Comparison of insect survival rates between oral and septic introduction of *S. marcescens* cells. Survival rates of male *R. pedestris* after oral infection **(A)** or septic injection **(B)** of *S. marcescens* Db11 strain (*n* = 30 insects per condition).

The survival rates of *R. pedestris* were measured after oral infection (Figure 1A) by feeding the cultured *S. marcescens* though the mouth, or after septic infection (Figure 1B) by injecting the cultured *S. marcescens* into hemolymph. In septic infection, approximately 50% of the insects injected with 100 *S. marcescens* cells died (half-maximal lethal dose [LD_50_] = 100 cells), and more than 80% of the insects died after septic injection of 600 cells in four days (Figure 1B). In comparison, most insects survived oral infection with 1.0 × 10^7^ cells (Figure 1A). The striking contrast in these results suggests that the potent virulence of cultured *S. marcescens* cells differs according to the route of infection.

**Figure 2:**
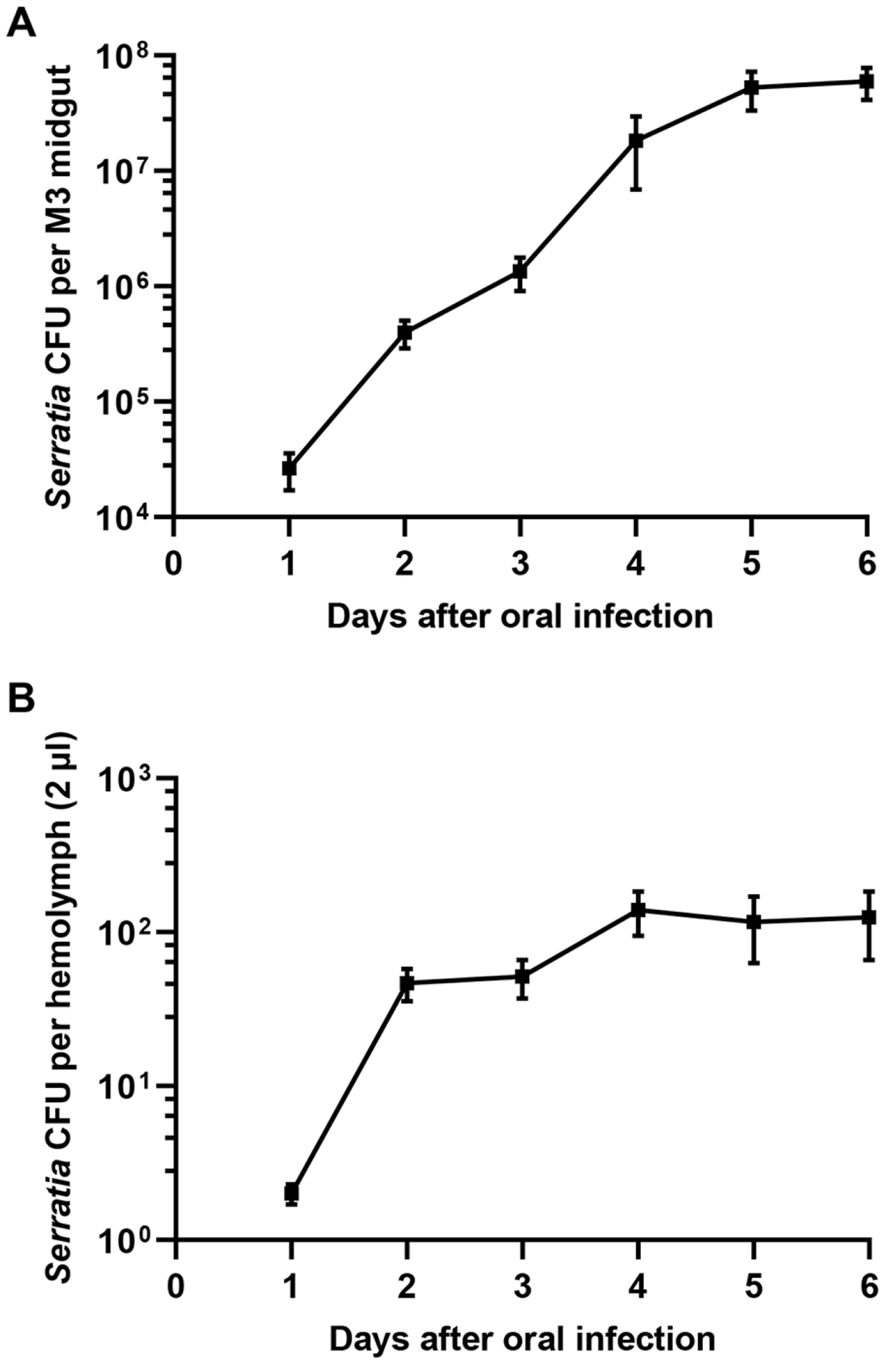
Titers of *S. marcescens* cells in the M3 midgut and hemolymph after oral infection. After oral infection with *S. marcescens*, bacterial titers in the M3 midgut **(A)** and hemolymph **(B)** were measured at intervals of one day.

Since the avirulence of orally infected *S. marcescens* cells may be due to bacterial clearance in the midgut, the titer of *S. marcescens* in the midgut of *R. pedestris* was investigated. Orally introduced *S. marcescens* cells were observed in the *R. pedestris* M3 midgut with a titer of more than 10^7^ cells per insect (Figure 2A). When *S. marcescens* cells from hemolymph were measured after oral infection, approximately 40–100 *S. marcescens* cells per 2 μL of hemolymph were recovered 2 days after infection (Figure 2B). These results suggest that orally infected *S. marcescens* cells can colonize and proliferate in the *R. pedestris* M3 midgut and then penetrate into the host hemolymph from the M3 midgut.

### Septic injection of the M3-colonizing *Serratia marcescens* cells fails to kill host insects and survive in the host hemolymph

Septic injection of *S. marcescens* into the hemolymph resulted in a high degree of insecticidal activity, with 100 cells (Figure 1B). However, a higher number of *S. marcescens* cells found in the hemolymph after oral infection showed no adverse effects on host insects (Figure 2B). Based on these results, we hypothesized that orally infected *S. marcescens* cells would lose their virulence in the host midgut. To test this hypothesis, we examined the pathogenicity of midgut-colonizing *S. marcescens*.

**Figure 3:**
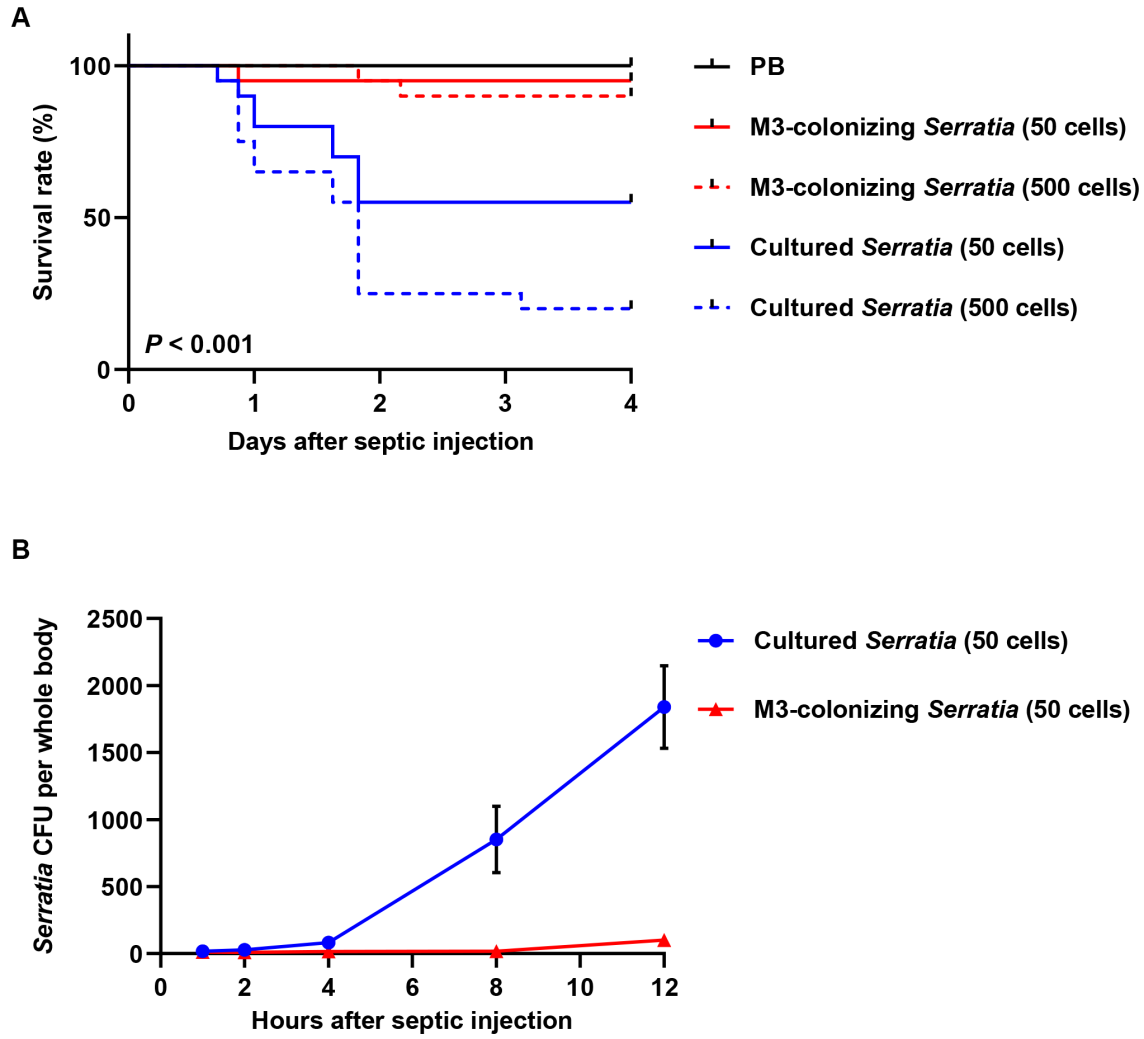
Insect survival rates and bacterial titers in hemolymph after septic infection with cultured or M3-colonizing *S. marcescens* cells. **(A)** Survival rates of male *R. pedestris* were measured after septic injection of the cultured or M3-colonizing *S. marcescens* cells. Approximate numbers of bacterial cells used for the infection are indicated on the graph (*n* = 30). This data is representative of three independent experiments. Log-rank (Mantel–Cox) test was used to assess the survival curve. **(B)** *S. marcescens* titers in hemolymph were measured at 1, 2, 4, 8, and 12 h post septic injection. Means and SEMs are indicated as data points and error bars, respectively (*n* = 10).

Because orally infected *S. marcescens* cells are colonized the M3 region, we collected midgut-colonizing *S. marcescens* cells by dissecting M3 midgut regions. Midgut-colonizing *S. marcescens* cells were septically injected into *R. pedestris*, and the survival rates of the host insects were measured. When the septic injections of the same numbers of M3-colonizing or cultured *S. marcescens* cells (50 and 500 cells/insect) were compared, approximately 90% of the insects injected with M3-colonizing *S. marcescens* cells survived, whereas only 50% of the insects injected with 50 cells and 20% injected with 500 cells of cultured *S. marcescens* survived (Figure 3A). We then measured colony forming units (CFUs) in the insect’s whole body at different time points after septic injection of M3-colonizing or cultured *S. marcescens* cells. While the number of CFUs reached almost 1,800 cells per insect 12 h after septic injection of cultured *S. marcescens* (50 cells), fewer than 100 CFUs were detected after septic injection of M3-colonizing *S. marcescens* cells (Figure 3B). These results show that cultured *S. marcescens* cells were able to overcome immune responses and proliferate in the host, consequently killing the host insects. However, M3-colonizing *S. marcescens* cells were susceptible to the host immune responses and cannot survive in the hemolymph.

### *Serratia marcescens* cells lose their LPS O antigens during colonization of the M3 midgut

**Figure 4:**
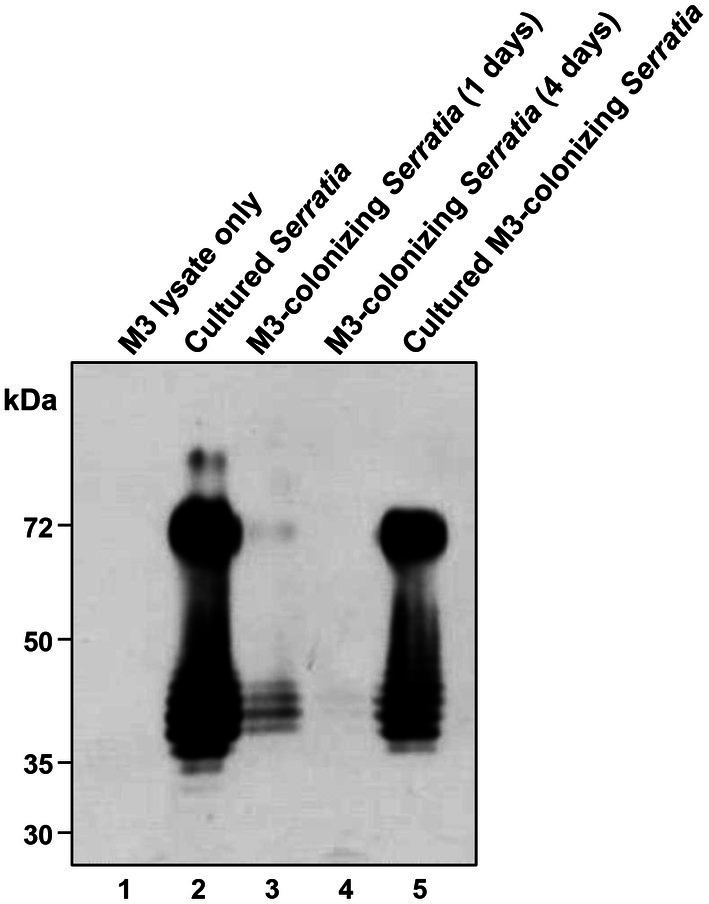
Western blot analysis of *S. marcescens* LPS O antigen. *S. marcescens* samples (10^7^ cells each) and a M3 midgut sample were subjected to SDS-PAGE. LPS O antigens separated in the gel were visualized by western blots using anti-*Serratia* O antigen antibodies. Lane 1, M3 lysate without bacterial oral infection; lane 2, *in vitro* cultured *S. marcescens*; lane 3, M3-colonizing *S. marcescens* at 1 day post oral infection; lane 4, M3-colonizing *S. marcescens* at 4 days post oral infection; lane 5, M3-colonizing *S. marcescens* after *in vitro* culturing for 4 days.

**Figure 5:**
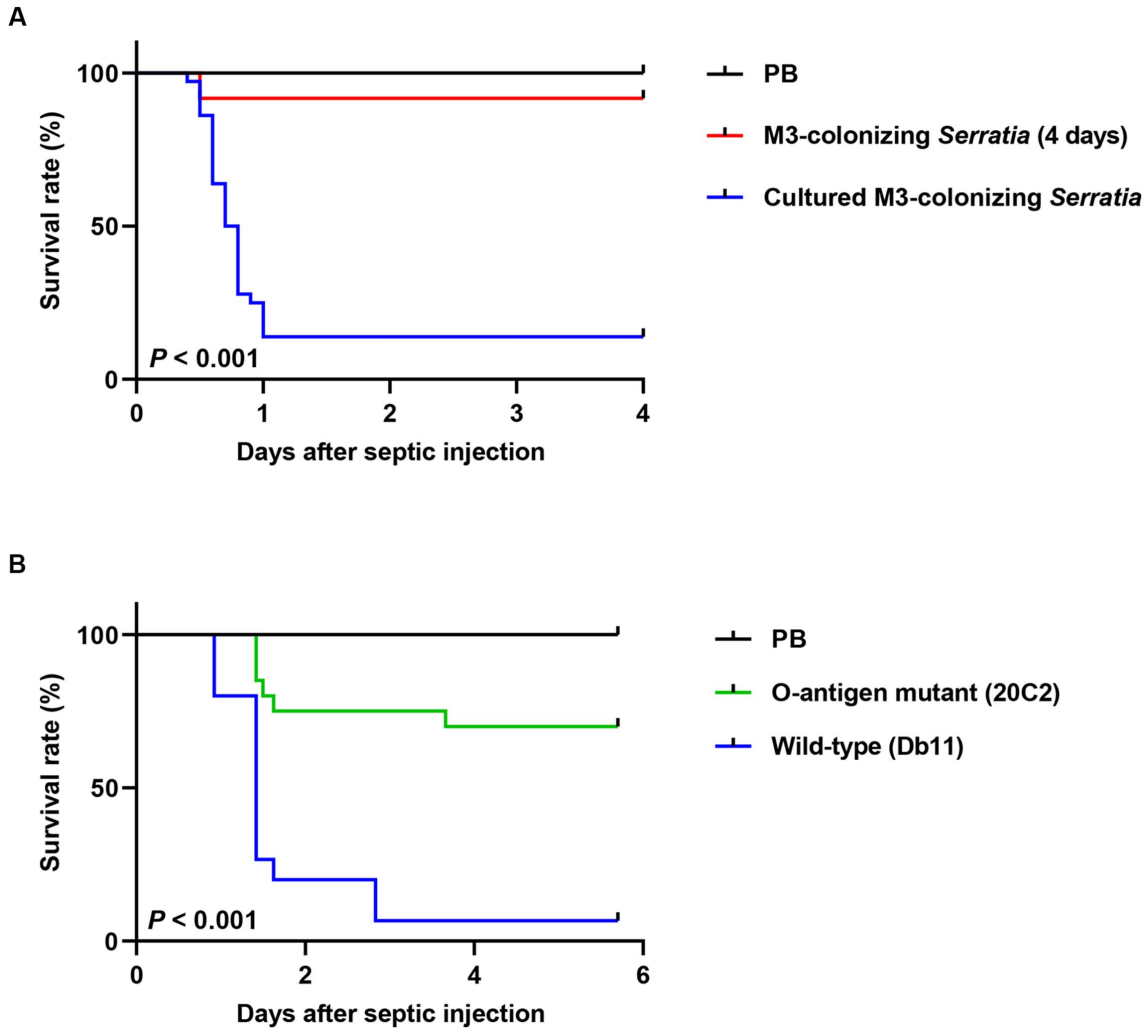
Comparison of insect survival rates after septic injection between the O antigen-deficient mutant and wild-type *S. marcescens* cells. **(A)** Survival rates of male *R. pedestris* were measured after septic injection of 100 cells of the M3-colonizing or cultured M3-colonizing *S. marcescens* cells (*n* = 30). **(B)** The survival rates of male *R. pedestris* were measured after septic injection of 200 cells of the O antigen-deficient mutant strain 20C2 or wild-type *S. marcescens* strain Db11 (*n* = 20). Data are representative of more than three independent experiments. Log-rank (Mantel–Cox) test was used to assess the survival curve.

In our previous study, we demonstrated that gut-symbiont *Burkholderia insecticola* cells lose their LPS O antigen after colonization in the symbiotic organ, M4 midgut of *R. pedestris* (Kim et al., 2015b). We speculated that M3-colonizing *S. marcescens* cells may also lose their LPS O antigens after colonizing the M3 midgut and therefore become susceptible to host immune responses. To test this possibility, the LPS patterns of 10^7^ M3-colonizing *S. marcescens* cells were analyzed using anti-*Serratia* O antigen antibodies (Figure 4, lanes 3 and 4). Non-infected M3 lysate (Figure 4, lane 1) was used as a negative control, and the same bacterial numbers of the cultured *S. marcescens* cells (Figure 4, lane 2) were used as O antigen-positive controls. As expected, the O antigen ladders of the cultured *S. marcescens* cells were recognized by anti-*Serratia* O antigen antibodies (Figure 4, lane 2), and no bands were observed in the M3 midgut lysate (Figure 4, lane 1). In comparison with the cultured cells, the O antigen ladders of M3-colonizing *S. marcescens* dramatically decreased from 1 day post-colonization (Figure 4, lane 3) and became almost undetectable at 4 days post-colonization (Figure 4, lane 4). When M3-colonizing *S. marcescens* cells were cultured, O antigen ladders were restored (Figure 4, lane 5). When comparing the insecticidal activity of M3-colonizing or cultured M3-colonizing *S. marcescens* cells (100 cells per insect), only 10% of insects injected with cultured M3-colonizing *S. marcescens* cells survived, showing restoration of pathogenicity of M3-colonizing *S. marcescens* through *in vitro* cultivation (Figure 5A). These results suggest that the pathogenicity of *S. marcescens* may be associated with the O antigen.

To prove that the loss of O antigens is a key cause of avirulence in M3-colonizing *S. marcescens*, we examined the virulence of the O antigen-deficient mutant strain 20C2 derived from the wild-type Db11 strain. The insect survival rates of the cultured *S. marcescens* Db11 and 20C2 strains after septic injection were compared (Figure 5B). When the O antigen-deficient 20C2 mutant strain (200 cells per insect) was septically injected into *R. pedestris*, 70% of the insects survived. However, with the wild-type Db11 strain, less than 5% of the insects survived, indicating that *S. marcescens* attenuates without the O antigen. This clearly shows that LPS O antigens contribute to the pathogenicity of *S. marcescens*.

### M3-colonizing *Serratia marcescens* cells were susceptible to cellular immune response of *Riptortus pedestris*

**Figure 6:**
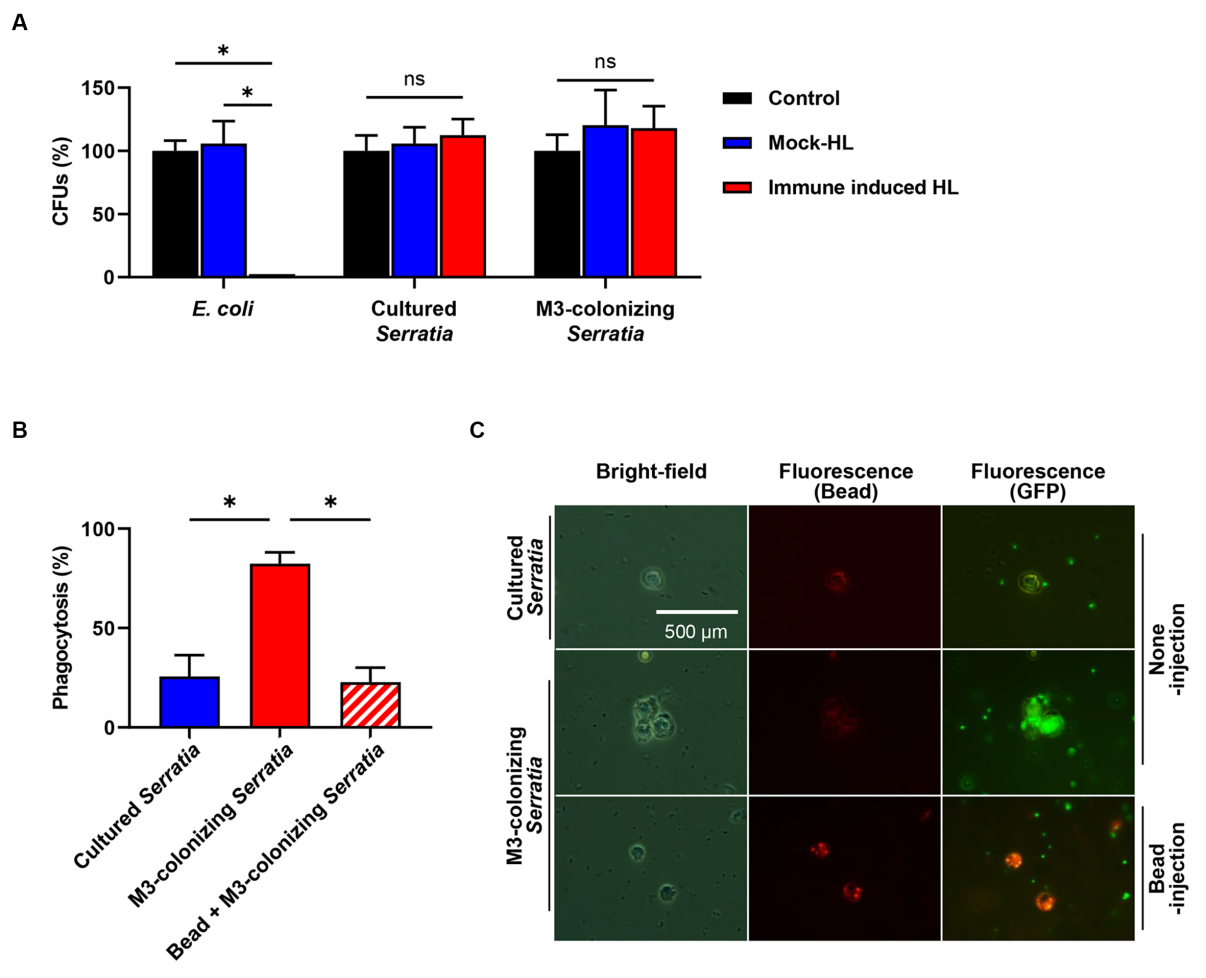
Susceptibility assays of the M3-colonizing *S. marcescens* cells against humoral and cellular immune responses of insect. **(A)** Bacterial susceptibility to male *R. pedestris* humoral immune responses was analyzed using hemolymph without an injection (control), hemolymph with a PB injection (mock HL), and hemolymph with *E. coli* injection (immune-induced HL). Means and standard deviations (SDs) are indicated as columns and error bars, respectively (*n* = 3). Asterisks indicate statistically significant differences (unpaired multiple *t-*test; NS, not significant; *p < 0.001). The data shown are representative of three independent experiments. **(B)** Susceptibility to cellular immune responses was analyzed by measuring percentages of *R. pedestris* immune cells phagocytizing GFP-expressing *S. marcescens*. To examine the phagocytosis of hemocytes, hemolymph was collected 3 h after injection of bacteria. Means and SDs are indicated as columns and error bars, respectively (*n* = 5). Asterisks indicate statistically significant differences (unpaired multiple *t-*test; *p < 0.001). The data shown are representative of three independent experiments. **(C)** Microscopic images show that phagocytosis occurred in M3-colonizing *S. marcescens* cells. Images with the same location were taken with bright-field microcopy to localize the hemocytes (left panels). Fluorescent microscopic images are showing localization of latex beads (middle panels) and GFP-expressing *S. marcescens* cells (right panels).

We investigated how O antigen-deficient M3-colonizing *S. marcescens* cells were cleared in the host hemolymph (Figure 3B) by humoral immunity, in which antimicrobial peptides play a major role, or by cellular immunity, in which phagocytic insect immune cells are key players. To test bacterial susceptibility to humoral immunity, M3-colonizing *S. marcescens* cells were treated with immune-induced hemolymph, which was collected from *E. coli*-challenged *R. pedestris*, heat-treated, and centrifuged to remove immune cells and large proteins. Hemolymph containing antimicrobial peptides that exhibited a bactericidal effect on *E. coli* did not reduce the number of CFUs of M3-colonizing *S. marcescens* cells (Figure 6A). In cellular immunity, less than 25% of *R. pedestris* hemocytes were observed with the cultured *S. marcescens* cells. However, with M3-colonizing *S. marcescens* cells, hemocytes effectively phagocytized bacteria, with more than 80% of immune cells engulfing M3-colonizing *S. marcescens* cells (Figures 6B,C). These results show that O antigen-deficient M3-colonizing *S. marcescens* cells are susceptible not to humoral immunity but to cellular immunity of *R. pedestris*.

### M3-colonizing *Serratia marcescens* cells restore their virulence when host cellular immunity is suppressed

**Figure 7:**
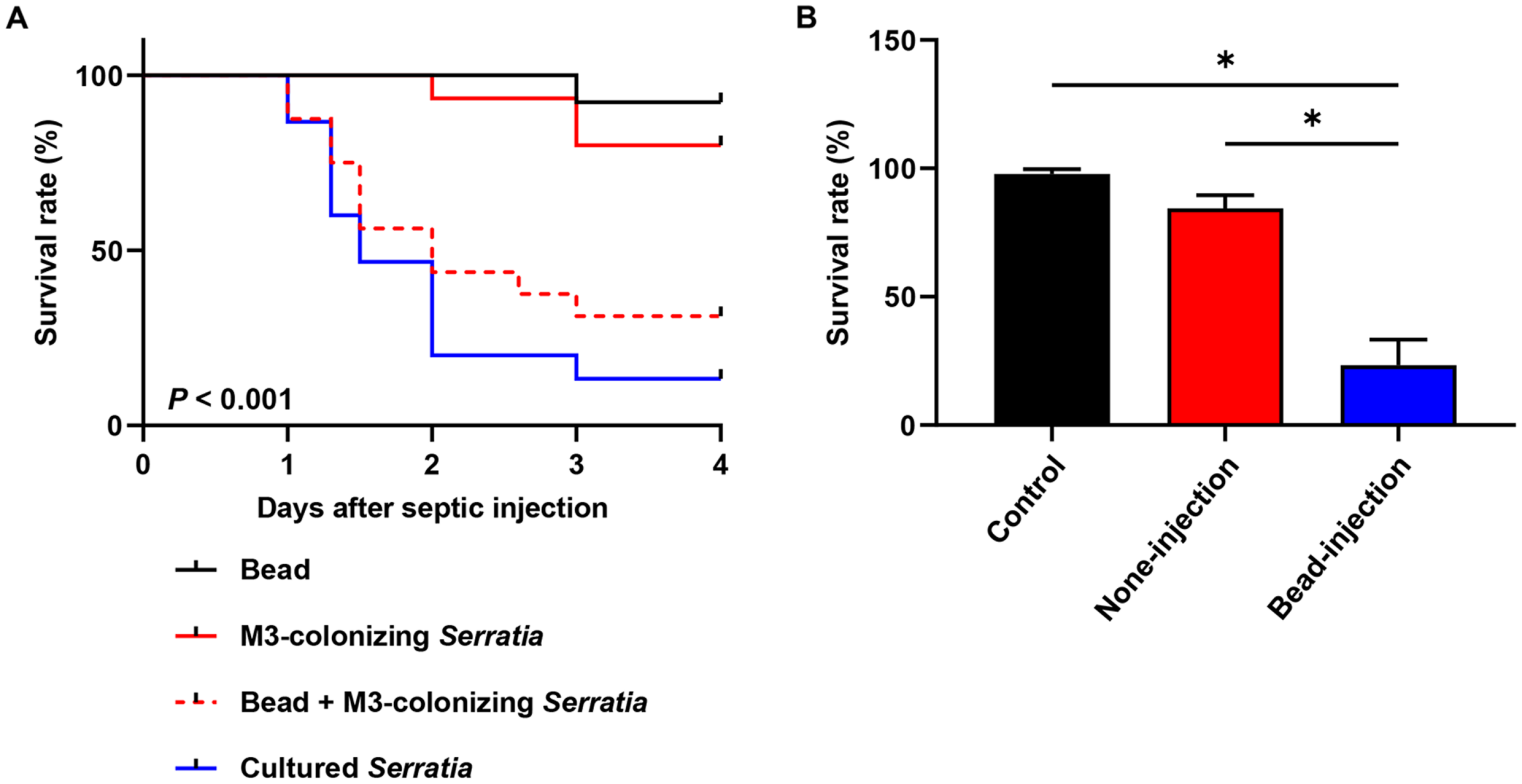
Pathogenicity of M3-colonizing *S. marcescens* cells in cellular immunity-repressed insects. **(A)** Insect survival rates were compared among injection with beads only, injection with cultured *S. marcescens* cells, injection with M3-colonizing *S. marcescens* cells, and co-injection with beads and M3-colonizing cells (*n* = 30). Suppression of cellular immunity was achieved by injecting beads (1.8 × 10^10^ beads/insect). For bacterial challenges, 1.0 × 10^3^ bacterial cells were septically injected into each insect. This data is representative of three independent experiments. Log-rank (Mantel–Cox) test was used to assess the survival curve. **(B)** Survival rates of cellular immune suppressed *R. pedestris* were measured after oral infection of 10^6^ cells/insect of the cultured *S. marcescens* cells (*n* = 30, 4 days post oral infection). Asterisks indicate statistically significant differences (unpaired multiple *t-*test; *p < 0.001). The data shown are representative of results from three independent experiments.

We postulated that orally infected *S. marcescens* cells alter the O antigen and become avirulent during colonization of the M3 midgut. Consequently, when they reach the hemolymph, M3-colonizing *S. marcescens* cells are effectively engulfed by host hemocytes before their virulence is restored. To verify this postulate, we suppressed host cellular immunity using latex microbeads and examined the restoration of virulence in M3-colonizing *S. marcescens* cells. Latex microbeads were injected into the hemolymph to saturate phagocytosis of immune cells. Bead-engulfed hemocytes were unable to phagocytize the M3-colonizing *S. marcescens* cells (Figures 6B,C). When a majority of insects (90%) septically infected with the M3-colonizing *S. marcescens* cells survived, only 25% of the insects survived from co-injection with beads and M3-colonizing *S. marcescens* cells after 3 days (Figure 7A). Suppression of the cellular immune response by the beads dramatically decreased the survival rates of *R. pedestris* insects challenged with M3-colonizing *S. marcescens* cells, implying the restoration of *S. marcescens* virulence. In addition, insects with suppressed cellular immunity by bead injection were more susceptible to oral infection of *S. marcescens* Db11 than insects without bead injection (Figure 7B). These results indicates that cellular immune responses are important for protection against the pathogenicity of O antigen-deficient M3-colonizing *S. marcescens* cells in the hemolymph.

## Discussion

It is questionable how insects handle orally-fed potent entomopathogen, *S. marcescens*, and how they survive from the attack of *S. marcescens* virulence factors. Here, bean bug *R. pedestris* was used as a host insect due to the availability of enough numbers of gut colonizing-*S. marcescens* cells. When the potent entomopathogen *S. marcescens* cells with an LD_50_ of 100 cells were introduced orally to *R. pedestris* insects, they did not induce pathogenicity to hosts even with a high number of cells (>10^8^) present in the M3 midgut and more than 100 cells in the hemolymph. The avirulence of gut-colonizing *S. marcescens* cells was confirmed by the high survival rates of insects after septic infection of M3-colonizing *S. marcescens* cells. M3-colonizing *S. marcescens* cells were resistant to humoral immunity but susceptible to cellular immunity-related phagocytosis of *R. pedestris* hemocytes. When the cellular immune response was artificially suppressed, the M3-colonizing *S. marcescens* cells showed insecticidal activity against *R. pedestris.* To elucidate the mechanisms responsible for the loss of virulence of orally infected *S. marcescens* cells, we focused on their susceptibility to phagocytosis and reversibility of virulence outside of midguts. Based on our previous study on the gut symbiont *Burkholderia*, we examined the LPS of gut-colonizing *S. marcescens* cells and found a loss of O antigens, which could be restored by *in vitro* culture. This indicates that alteration of LPS O antigens in *S. marcescens* is a critical mechanism in the loss of virulence in gut-colonizing *S. marcescens* cells.

LPS is a major surface component of the outer membrane of gram-negative bacteria and is structurally composed of three regions: lipid A, core oligosaccharide, and O antigen. It is important for maintenance of physiological properties such as cell integrity, permeability, and the secretion of extracellular toxins (Raetz and Whitfield, 2002; Caroff and Karibian, 2003; Herlax et al., 2005; Ishii et al., 2014; Bertani and Ruiz, 2018). The innermost lipid A is a membrane-anchored hydrophobic region that connects to a core oligosaccharide via 2-keto-3-deoxyoctonate (KDO) units. The core oligosaccharide is then linked to the outermost region of the O antigen, which consists of repeating oligosaccharide units. The O antigen provides protection against environmental and immunological factors and is closely associated with bacterial pathogenicity (Orskov et al., 1977; Raetz and Whitfield, 2002). In pathogenesis, the O antigen is an important virulence factor that directly interacts with host components and protects bacterial cell membranes from host’s membrane-attacking compounds (Trent et al., 2006). Although the O antigen may play various roles in different bacteria, O antigen-deficient bacteria are reportedly susceptible to serum complements and antimicrobial peptides, and are therefore much less efficient at invading and surviving in hosts (Van Den Bosch et al., 1997; Burns and Hull, 1998; Nesper et al., 2001; Murray et al., 2006; Gunn and Ernst, 2007; Kim et al., 2016). The O antigen is also involved in evading cellular immune responses by inhibiting phagocytosis. In the case of the fish pathogen *Vibrio anguillarum*, the O antigen is used to colonize skin tissue by evading phagocytosis by epithelial cells (Lindell et al., 2012). When the effect of the O antigen on the phagocytic activity of mouse macrophages was investigated, *E. coli* or *Burkholderia cenocepacia* strains lacking the O antigen were more effectively internalized than the wild type (Eder et al., 2009; Saldías et al., 2009).

Previous reports on the role of *S. marcescens* LPS support our finding that alteration of O antigen is involved in avirulence of orally introduced *S. marcescens*. A transposon mutation of the *wecA* gene, which is involved in the biosynthesis of LPS O antigen, suppresses apoptosis of immune cells of the silkworm *Bombyx mori* insect by *S. marcescens* (Ishii et al., 2012). A culture supernatant of the *wecA* mutant strain showed reduced hemolymph bleeding activity compared to that of wild-type *S. marcescens*, suggesting that the O antigen contributes to the production of bleeding factors (Ishii et al., 2014). In addition, *S. marcescens* lacking O antigens showed significantly lower cytotoxicity in *C. elegans* than *S. marcescens* with O antigen (Kurz et al., 2003). The smooth strains of *S. marcescens* with O antigen showed a 20–30-fold increase in hemolytic activity compared with rough strains without O antigen (Poole and Braun, 1988). These reports indicate that the loss of O antigens results in avirulence of *S. marcescens*.

In a symbiosis study of *R. pedestris*, we showed that orally infected *Burkhoderia* cells lose their O antigens after colonization of the M4 midgut region of the host *R. pedestris* (Kim et al., 2015b). The O antigens of *Burkhoderia* cells play a role in the initial symbiotic association with *R. pedestris*, in which bacterial resistance to host immune responses is required for survival before reaching the M4 midgut (Kim et al., 2016). However, once they have colonized the M4 midgut, the O antigens are lost in the gut symbiont. O antigen-deficient *Burkholderia* cells are highly susceptible to host-derived antimicrobial peptides, such as riptocin, defensin, and thanatin, and the M4 midgut suppresses the expression of these antimicrobial peptides so that gut symbionts can persist in the M4 midgut (Kim et al., 2015b; Lee et al., 2022).

It remains unclear how the entomopathogen *S. marcescens* and the beneficial symbiont *Burkholderia* lose their O antigens while colonizing the midgut of *R. pedestris*. From the host’s perspective, alteration of bacterial LPS allows the host to easily manage gut-colonizing bacteria, both symbionts and pathobionts. From the bacterial perspective, gut-colonizing bacteria can conserve energy by controlling unnecessary cell wall components that are not directly related to survival in the host gut environment. An investigation of the molecular mechanism of O antigen alteration in gut-colonizing bacteria may provide insight into the host’s control of gut microbiota and adaptation of gut microbiota in hosts.

## Data availability statement

The original contributions presented in the study are included in the article/supplementary material, further inquiries can be directed to the corresponding author.

## Author contributions

JL: Data curation, Funding acquisition, Investigation, Writing – original draft, Writing – review & editing. JUK: ata curation, Investigation, Writing – review & editing. BLL: Investigation, Supervision, Writing – review & editing. JKK: Conceptualization, Data curation, Funding acquisition, Investigation, Supervision, Validation, Writing – original draft, Writing – review & editing.
